# Protocol to uncover the protein interactome of small non-coding vault RNAs through RNA antisense purification coupled to mass spectrometry

**DOI:** 10.1016/j.xpro.2026.104414

**Published:** 2026-03-06

**Authors:** Sander B. van der Kooij, Jorn E. Stok, Annemarthe G. van der Veen

**Affiliations:** 1 Department of Immunology, Leiden University Medical Centre, Leiden, the Netherlands

**Keywords:** Cell biology, Immunology, Mass spectrometry, Molecular biology

## Abstract

Vault RNAs (vtRNAs) are a family of four small non-coding RNAs (ncRNAs). Here, we present a protocol to identify protein interactors of vtRNA1-1, vtRNA1-2, and vtRNA1-3 in human lung epithelial cells through RNA antisense purification coupled to mass spectrometry (RAP-MS). We describe steps for using biotinylated vtRNA-specific antisense DNA probes to isolate vtRNA-protein complexes that are covalently crosslinked by treatment with ultraviolet (UV) light, followed by mass spectrometry-based analysis. This protocol can be adapted to other cells or small ncRNAs.

For complete details on the use and execution of this protocol, please refer to Stok et al.^1^

## Highlights

• Steps to identify protein interactome of vault RNAs during infection with EMC virus
• Guidelines on how to design an optimal probe for targeting small non-coding RNAs
• Steps for performing RNA antisense purification coupled to mass spectrometry
• Guidance on how to interpret and analyze the obtained mass spectrometry data

## Abstract (editor highlights)

Publisher’s note: Undertaking any experimental protocol requires adherence to local institutional guidelines for laboratory safety and ethics.

## Graphical abstract

**Figure:**
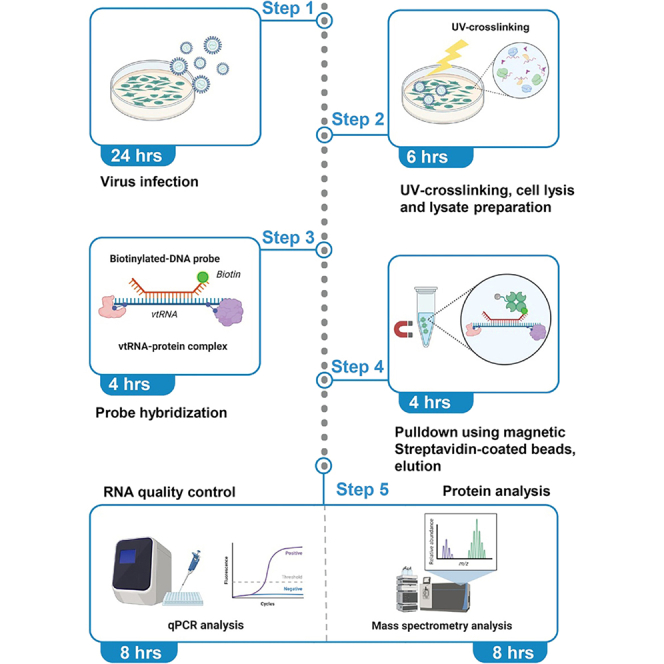

## Before you begin

Small ncRNAs are typically less than 200 nucleotides (nt) in length and are not translated into proteins. Instead, they perform regulatory function in cells. The vtRNA family consists of four members, namely vtRNA1-1, vtRNA1-2, vtRNA1-3, and vtRNA2-1, which range between 88 and 140 nt in length.^2^ VtRNAs are implicated in diverse cellular processes, such as autophagy, apoptosis, and immune activation.^3^^,^^4^^,^^5^ The expression of vtRNAs is often increased upon cellular stress, including viral infection, but their precise cellular function is not well defined.^6^ RNA-protein interaction studies may shed light on their intracellular function, but the capture of proteins that directly and specifically interact with such small ncRNAs is complex and requires specialized protocols. In the past decades, several tools have been developed to study the protein interactome of ncRNAs.^7^ This includes RNA antisense purification coupled to mass spectrometry (RAP-MS), which allows the identification of the interactome of ncRNAs through pulldown of a specific RNA species, using a biotinylated, antisense DNA probe that is complementary to the target of interest and immobilized on streptavidin beads.^8^ In addition, RAP-MS uses UV-light for covalent crosslinking of short-range interactions within RNA-protein complexes, allowing for stringent and highly denaturing purification conditions to minimize background and non-specific binders and to specifically capture crosslinked proteins.^8^ This method was first developed for the identification of high-confidence protein interactors of *long* ncRNAs by Colleen McHugh and Mitchell Guttman in 2015 and has been used by several other groups for this purpose.^8^^,^^9^^,^^10^^,^^11^ We have used this protocol to retrieve *small* ncRNAs, namely vtRNAs, from cells in the absence or presence of a replicating virus to study how viral infection shapes the vtRNA interactome.^1^

Here, we outline the different steps involved in executing the RAP-MS protocol, based on the protocol of McHugh et al., 2015 with minor modifications. The protocol is designed around pulldowns of vtRNA1-1, vtRNA1-2, and vtRNA1-3 from the human lung epithelial cell line A549 in the absence or presence of encephalomyocarditis virus (EMCV). A comparable experimental setup can be used for other similar-sized ncRNAs, viruses or cell lines. The method describes the design of the biotinylated antisense DNA probes used for the pulldown. Subsequently, a step-by-step method is provided to execute the RAP-MS protocol. Finally, we provide insight into the analysis and interpretation of the data obtained through mass spectrometry. Prior to the start of the protocol, the probes should be designed and ordered and the buffers and materials as discussed in the “Key resource table” and “Material and Methods” should be prepared.

### Innovation

This protocol differs from earlier protocols in the design and use of a *single* antisense DNA probe for hybridization to a small ncRNA, as opposed to the use of *multiple* probes for a single RNA target as described by McHugh et al., 2015. In a conventional RAP-MS setup, a tiled approach is employed that allows the usage of multiple 90 nt long probes, hybridizing to different regions of the target RNA. However, due to their short length, small ncRNAs, including vtRNAs, do not allow such as design. We therefore used a single probe targeting an individual vtRNA, designed in a way to minimize complementarity to other vtRNAs, which are highly homologous to each other. As the use of a single probe increases the chance to retrieve non-specific interactors, a secondary validation method using a protein-centric method, such as RNA immunoprecipitation followed by qPCR (RIP-qPCR) is recommended to validate the results of the RAP-MS.^12^

### Probe design

For vtRNA1-1, vtRNA1-2, and vtRNA1-3, a single biotinylated *antisense* DNA probe complementary to the respective vtRNA was designed, ensuring minimal sequence homology between the probes. The probes were designed around the middle section of the vtRNA, as these sections are less homologous. Subsequently, the probes were trimmed to 80 nt (instead of the canonical 90 nt length) to avoid the highly homologous 5′ and 3′ ends of the vtRNAs. However, due to the strong homology between vtRNA1-1, vtRNA1-2 and vtRNA1-3, complete specificity is impossible. The 80 nt long probes can be ordered through Integrated DNA Technologies (IDT) and should be modified with biotin at the 5′ end and purified through HPLC. As a negative control, the reverse complement of the vtRNA-interacting probe (i.e., a “*sense* probe”) was used. Of note, as vtRNAs contain regions of intramolecular complementarity, sense probes may unintentionally interact with the target RNA, leading to potential false negatives that are excluded during data analysis. To circumvent this, we recommend using an additional, non-related (or scrambled) DNA probe of similar length.

**Table:** 

Probe identifier	Probe sequence
vtRNA1-1 (sense)	/5Biosg/GC GGT TAC TTC GAC AGT TCT TTA ATT GAA ACA AGC AAC CTG TCT GGG TTG TTC GAG ACC CGC GGG CGC TCT CCA GTC CTT
vtRNA1-1 (antisense)	/5Biosg/AA GGA CTG GAG AGC GCC CGC GGG TCT CGA ACA ACC CAG ACA GGT TGC TTG TTT CAA TTA AAG AAC TGT CGA AGT AAC CGC
vtRNA1-2 (sense)	/5Biosg/CTT TAG CTC AGC GGT TAC TTC GAG TAC ATT GTA ACC ACC TCT CTG GGT GGT TCG AGA CCC GCG GGT GCT TTC CAG CTC TT
vtRNA1-2 (antisense)	/5Biosg/AAG AGC TGG AAA GCA CCC GCG GGT CTC GAA CCA CCC AGA GAG GTG GTT ACA ATG TAC TCG AAG TAA CCG CTG AGC TAA AG
vtRNA1-3 (sense)	/5Biosg/TTT AGC TCA GCG GTT ACT TCG CGT GTC ATC AAA CCA CCT CTC TGG GTT GTT CGA GAC CCG CGG GCG CTC TCC AGC CCT CT
vtRNA1-3 (antisense)	/5Biosg/AGA GGG CTG GAG AGC GCC CGC GGG TCT CGA ACA ACC CAG AGA GGT GGT TTG ATG ACA CGC GAA GTA ACC GCT GAG CTA AA

### Quality control considerations

To successfully perform this protocol, we recommend the following quality control measurements. First, if relevant, productive viral infection and replication should be determined by measuring the abundance of EMCV transcripts by qPCR, normalized to a housekeeping gene (e.g., 18s rRNA or GAPDH), based on the ΔΔCt method. Similarly, increased vtRNA1-1, vtRNA1-2 and vtRNA1-3 expression upon EMCV infection can be analyzed by qPCR.^1^ The *“input”* samples taken from mock versus infected conditions can be analyzed as described in the step “RNA extraction, cDNA synthesis and qPCR”.

Secondly, to determine the pulldown efficiency of the target RNA, the original RAP-MS protocol indicates to measure target abundance in *“input + probe”, “unbound”* and *“pulldown”* samples using qPCR. The enrichment of the target within the *“pulldown”* samples versus the “*input + probe*” samples is a direct measure of pulldown efficiency. Alternatively, the relative depletion of the target in the “*unbound”* versus the “*input + probe*” sample may serve as an indirect measurement. This analysis is challenging in the case of vtRNA pulldowns due to the fact that the 80 nt long DNA probes are nearly covering the complete vtRNA transcripts. The DNA probes in the resulting DNA:RNA hybrids are poorly digested by TURBO DNase, leading to carry-over of the probe into the cDNA samples and overestimation of RNA abundance, as residual DNA probe can serve as a template in qPCR. This results in high signal intensities that obscure accurate quantification of depletion or enrichment. This limitation is inherent to the small size of the vtRNAs (<100 nt) combined with the 80 nt long DNA probe, excluding primer design that does not overlap with the region targeted by the probe. One may consider to shorten the probe length, at the expense of specificity. Alternatively, one may choose to use Northern blot, which allows more stringent methods to reduce the binding of the antisense DNA probe to the vtRNA probe, to determine the pulldown efficiency.^13^

## Key resources table

**Table:** 

REAGENT or RESOURCE	SOURCE	IDENTIFIER
**Bacterial and virus strains**		

EMCV	Gift from Caetano Reis e Sousa, The Francis Crick Institute, London, UK	Ruckert strain

**Chemicals, peptides, and recombinant proteins**		

Tris-HCl pH 7.5	Thermo Fisher Scientific	15567–027
Tris-HCl pH 8.0	Thermo Fisher Scientific	15568025
Lithium chloride	Sigma-Aldrich	L7026-100ML
Dodecyl maltoside	Sigma-Aldrich	D4641
Sodium dodecyl sulphate	Thermo Fisher Scientific	AM9820
Sodium deoxycholate	Sigma-Aldrich	D6750
MgCl_2_	Thermo Fisher Scientific	AM9530G
CaCl_2_	Thermo Fisher Scientific	J63122.AD
EDTA pH 8.0	Thermo Fisher Scientific	AM9260G
Urea	Thermo Fisher Scientific	29700
TCEP	Thermo Fisher Scientific	77720
N-lauroylsarcosine sodium salt	Sigma-Aldrich	61747
Iodoacetamide	Thermo Fisher Scientific	A39271
Trypsin (TPCK-treated)	Thermo Fisher Scientific	20233
UltraPure Water	Thermo Fisher Scientific	10977049
Ethanol	Sigma-Aldrich	493511-62L
EGTA	Thermo Fisher Scientific	J60767.AD
cOmplete protease inhibitor	Roche	11836153001
RNasin (RNase inhibitor)	Promega	N2111
TURBO DNase	Invitrogen	AM2238
TURBO DNase buffer	Invitrogen	AM2238
RLT buffer	Qiagen	79216
UltraPure™ BSA	Thermo Fisher Scientific	AM2616
GENIUS nuclease (Benzonase)	Santa Cruz	sc-202391
Proteinase K	Thermo Fisher Scientific	25530049
Dithiothreitol (1M)	Thermo Fisher Scientific	P2325

**Critical commercial assays**		

Pierce BCA protein assay kit	Thermo Fisher Scientific	23225
SilverQuest silver staining kit	Thermo Fisher Scientific	LC6070

**Deposited data**		

RAP-MS dataset (analyzed)	This paper	N/A
RAP-MS dataset (raw data)	Stok et al., 2025^1^	ProteomeXchange Consortium, PXD055065

**Experimental models: cell lines**		

Human: A549	Gift from Rob Hoeben, LUMC, Leiden, The Netherlands	N/A

**Oligonucleotides**		

vtRNA1-1 Fw: TTAGCTCAGCGGTTACTTCGACAGTTC	Horos *et al.*,^4^ Cell, 2019	N/A
vtRNA1-1 Rv: AAAAGGACTGGAGAGCGCCC	Horos *et al.*,^4^ Cell, 2019	N/A
vtRNA1-2 Fw: GGCTGGCTTTAGCTCAGCGG	Horos *et al.*,^4^ Cell, 2019	N/A
vtRNA1-2 Rw: AAAAGAGCTGGAAAGCACCC	Horos *et al.*,^4^ Cell, 2019	N/A
vtRNA1-3 Fw: AGCGGTTACTTCGCGTGTCATC	Horos *et al.*,^4^ Cell, 2019	N/A
vtRNA1-3 Rv: AAGAGGGCTGGAGAGCGCC	Horos *et al.*,^4^ Cell, 2019	N/A
EMCV L region Fw: GCGCACTCTCTCACTTTTGA	N/A	N/A
EMCV L region Rv: TCGAAAACGACTTCCATGTCT	N/A	N/A

**Software and algorithms**		

Perseus 2.0.10.0	MaxQuant	N/A
Proteome Discoverer software (2.5)	Thermo Fisher Scientific	N/A

**Other**		

Eppendorf DNA LoBind tube 50 mL	Fisher Scientific	15581312
Eppendorf DNA LoBind tube 15 mL	Fisher Scientific	15571312
Eppendorf DNA LoBind tube 2.0 mL	Fisher Scientific	10031282
Eppendorf DNA LoBind tube 1.5 mL	Fisher Scientific	10051232
UVP Crosslinker (Analytik Jena)	Analytik Jena	CL-3000
Branson 2510 E-MT Ultrasonic bath	Branson	N/A
DynaMag™-PCR Magnet	Thermo Fisher Scientific	492025
DynaMag™-2 Magnet	Thermo Fisher Scientific	12321D
DynaMag™-15 Magnet	Thermo Fisher Scientific	12301D
Alpha 204 LCS plus (freeze dryer)	Martin Christ	N/A
4–15% Mini-PROTEAN® TGX™ Precast Protein Gels	Biorad	4561083
Streptavidin-coated magnetic beads	NEB	S1420S
SuperScript IV VILO Master Mix	Thermo Fisher Scientific	11756050
SYBR™ Green Universal Master Mix (with rox)	Thermo Fisher Scientific	4312704
Eppendorf Thermomixer C	Eppendorf	5382000066
Proteineer DP digestion robot	Burker	N/A
Cell lifter	Corning	3008
15 cm cell culture dish	Greiner	639160
DMEM	Thermo Fisher Scientific	41966029
FCS	Capricorn	FBS-11a
PBS	Thermo Fisher Scientific	10010023
27G Needle	BD	305770
NUWIND-C200RE High-speed refrigerated microcentrifuge (>16.000 g)	NuAire	NU-C200R-E
Liquid nitrogen	N/A	N/A
Dynabeads™ MyOne™ Silane	Thermo Fisher Scientific	37002D
Exploris480 mass spectrometer	Thermo Fisher Scientific	N/A
Minimal human UniProt proteome	UniProt	UP000005640
EMCV polyprotein (Ruckert Strain) UniProt proteome	UniProt	UP000002319

## Materials and equipment

The given buffer recipes are sufficient to execute the protocol as described, except when stated otherwise.

**Table:** Total Cell Lysis Buffer

Reagent	Final concentration	Amount
Tris-HCl pH 7.5 (1 M)	10 mM	400 μl
Lithium chloride (8 M)	500 mM	2500 μl
Dodecyl maltoside (20%)	0.5%	1000 μl
Sodium dodecyl sulphate (20%)	0.2%	400 μl
Sodium deoxycholate (12.5%)	0.1%	320 μl
UltraPure water	–	35.38 mL
**Total**	N/A	**40 mL**

Store at RT, make 1 day in advance.

**CRITICAL:** SDS is toxic in powder form, handle this compound in a fume hood.

**Table:** 200x DNase Salt buffer

Reagent	Final concentration	Amount
MgCl_2_ (1 M)	500 mM	500 μl
CaCl_2_ (1 M)	100 mM	100 μl
UltraPure water	–	400 μl
**Total**	–	**1 mL**

Store at RT, make 1 day in advance.

**Table:** 1.5x Hybridization Buffer (Prepare 2 tubes with 45 mL/tube)

Reagent	Final concentration	Amount
Tris-HCl pH 7.5 (1 M)	15 mM	672 μl
EDTA pH 8.0 (0.5 M)	7.5 mM	672 μl
Lithium chloride (8 M)	750 mM	4219 μl
Dodecyl maltoside (20%)	0.75%	1687.5 μl
Sodium dodecyl sulphate (20%)	0.3%	672 μl
Sodium deoxycholate (12.5%)	0.15%	540 μl
Urea	6 M	16.2 g
TCEP (0.5 M)	3.75 mM	337.5 μl
UltraPure water	–	36.2 mL
**Total**	–	**45 mL**

Store at RT, make 1 day in advance.

**CRITICAL:** SDS is toxic in powder form, handle this compound in a fume hood.

**Table:** 1x Hybridization Buffer (Prepare 2 tubes with 30 mL/tube)

Reagent	Final concentration	Amount
Tris-HCl pH 7.5 (1 M)	10 mM	300 μl
EDTA pH 8.0 (0.5 M)	5 mM	300 μl
Lithium chloride (8 M)	500 mM	1875 μl
Dodecyl maltoside (20%)	0.5%	750 μl
Sodium dodecyl sulphate (20%)	0.2%	300 μl
Sodium deoxycholate (12.5%)	0.1%	240 μl
Urea	4 M	7.2 g
TCEP (0.5 M)	2.5 mM	150 μl
UltraPure water	–	26.085 mL
**Total**	–	**30 mL**

Store at RT, make 1 day in advance.

**CRITICAL:** SDS is toxic in powder form, handle this compound in a fume hood.

**Table:** Benzonase Elution Buffer

Reagent	Final concentration	Amount
Tris-HCl pH 8.0 (1 M)	20 mM	20 μl
N-lauryl sarcosine (20%)	0.05%	2.5 μl
MgCl_2_ (1 M)	2 mM	2 μl
TCEP (0.5 M)	0.5 mM	1 μl
UltraPure water	–	974.5 μl
**Total**	–	**1 mL**

Store at RT, make 1 day in advance.

**Table:** NLS elution buffer

Reagent	Final concentration	Amount
Tris-HCl pH 8.0 (1 M)	20 mM	20 μl
EDTA pH 8.0 (1 M)	10 mM	20 μl
N-lauryl sarcosine (20%)	2%	100 μl
TCEP (0.5 M)	2.5 mM	5 μl
UltraPure water	–	855 μl
**Total**	–	**1 mL**

Store at RT, make 1 day in advance.

**Table:** 4x Protein sample buffer

Reagent	Final concentration	Amount
Tris-HCl pH 6.8 (1 M)	60 mM	60 μl
Sodium dodecyl sulphate (20%)	1.5%	75 μl
Glycerol (50%)	10%	200 μl
DTT (1 M)	15 mM	15 μl
UltraPure water	–	650 μl
**Total**	–	**1 mL**

Store at RT, make 1 day in advance.

**Table:** TURBO DNase elution Buffer

Reagent	Final concentration	Amount
10X TURBO DNase Buffer	1X	2 μl
TURBO DNase	1X	3 μl
RNasin (40 U/μl)	2 U/μl	1 μl
UltraPure water	–	14 μl
**Total**	–	20 μl

Store at 4 °C, make fresh immediately before use.

## Step-by-step method details

### Seeding of cells

**Timing: 1 h**

In this step, cells are seeded 24 hours prior to viral infection to allow the cells to attach overnight. Subsequently, the cells are mock-infected (with virus-free inoculum) or infected with EMCV for 24 hours.

1. Seed the A549 WT cells in a 15-cm dish aiming for a density of approximately 80–90% after overnight attachment of the cells.

***Note:*** EMCV is a lytic virus, therefore, to compensate for cell death, seed 3 dishes for the infected condition and 2 dishes for the uninfected condition. Further recommendations are discussed in “Problem 1”.

### Infection of seeded cells

**Timing: 2 h**

2. After 24 hours, inoculate the cells with EMCV at MOI 0.25.
  a. Aspirate the medium and replace with either the mock inoculum or the viral inoculum (9 mL), consisting of DMEM without FCS, to allow attachment of the virus to the cells.
  b. Incubate at 37 °C at 10% CO_2_ and gently rock the dish every 15 minutes.
  c. After 1 hour of incubation, add 9 ml of DMEM containing 20% FCS to each plate.

***Note:*** Handle EMCV at the appropriate biosafety level.

### Harvesting of infected cells and preparation for cell lysis

**Timing: 1 h**

In this step, cells are UV-treated to covalently crosslink the RNA and their protein interactors, and subsequently snap-frozen.

**CRITICAL:** All subsequent experimental steps should be performed on ice and in RNase-free conditions (reagents, filter tips) to maintain optimal RNA integrity.

**CRITICAL:** Use DNA LoBind tips and collection tubes to minimize RNA and protein loss due to non-specific binding to the tips and tubes.

**CRITICAL:** Work with freshly opened buffers and clean all equipment and bench spaces to minimize contamination of the samples with proteins such as BSA and common skin proteins (for example keratin).

3. After 24 hours of incubation, aspirate the medium and wash the cells with 10 ml ice-cold PBS.
4. Next, add 10 ml of ice-cold PBS prior to UV-crosslinking.
5. Irradiate cells in the UVP crosslinker with UV light (254 nm) at 0.8 J/cm^2^. Afterwards, return the plate to ice.

***Note:*** Pre-warm the UV bulb prior to step 2 by running an empty program at 0.3 J/cm^2^ to ensure equal crosslinking between all conditions.

6. Scrape the cells from the plate with a cell lifter, collect cells in a DNA LoBind 50 ml tube, and centrifuge 5 min at 1000 *g* at 4 °C.
7. Carefully aspirate the supernatant, snap-freeze the pellet on dry-ice, and store at −80 °C.

***Note:*** Cell pellets can be stored at −80°C for a maximum of 1 month.

### Cell lysis

**Timing: 3 h**

In this step, the snap-frozen cells are lysed and prepared for incubation with the hybridization probes.

8. Lyse each frozen cell pellet (uninfected and infected) in 5.4 ml total Cell Lysis buffer.
  a. Add Roche cOmplete protease inhibitor to a final concentration of 1x and RNase inhibitor (156 U/mL) and incubate for 15 minutes on ice.
  b. During the incubation, pass the lysate 3–5 times through a 27G needle.
9. Sonicate the samples with a microtip intensity of 15% for 2 minutes (20 seconds on, 20 seconds off) to ensure total cell lysis.
10. Add 200x DNase Salt buffer to a final concentration of 1x and add 20 U/mL of TURBO DNase, incubate for 10 min in a heat block at 37 °C.
11. Return the samples to ice and add the following compounds:

**Table:** 

Compound (concentration)	Final concentration in lysate
EDTA (0.5 M)	10 mM
EGTA (0.5 M)	5 mM
TCEP (0.5 M)	2.5 mM

12. Perform a BCA assay according to manufacturer’s instructions.

**CRITICAL:** Ensure an equal protein concentration between the uninfected and infected conditions, with at least 3 mg of protein per vtRNA pulldown.

13. Mix the lysate with 2X the sample volume of 1.5X Hybridization buffer and incubate on ice for 10 min.
14. Centrifugate the lysates at 16,000 *g* for 10 min at 4 °C to remove non-lysed cells and cellular debris.
15. Transfer the supernatant to a new DNA LoBind 50 ml tube and snap-freeze the supernatant in liquid nitrogen. Store at −80 °C.

***Note:*** Cell lysates can be stored at −80°C for a maximum of 1 month.

### Preparation of beads for capture of the probe

**Timing: 1 h**

In this part of the protocol the streptavidin-coated beads are prepared so that they can be used to recover crosslinked-RNA protein complexes via the biotinylated antisense probe.

16. Prepare 300 μl of streptavidin-coated magnetic beads for each pulldown.

***Note:*** 100 μl will be used for pre-clearing of the lysate and 200 μl will be used for pulldown of the probe.

17. Wash the beads four times with 900 μl 10 mM Tris-HCl pH 7.5 and twice with 900 μl 1x Hybridization buffer.

***Note:*** Washing of magnetic beads is performed with the DynaMag-2 Magnet, according to manufacturer’s instructions.

18. Resuspend the beads in 300 μl 1x Hybridization buffer for each pulldown, supplement with 1% BSA (final concentration of 0.01 mg/ml).
19. Block for 16 hours with end-over-end rotation at 4 °C.

***Note:*** For this protocol, two conditions (mock and infected) with each six pulldowns (sense and antisense for each respective vtRNA) are performed, therefore 3.6 mL of beads is used.

### Pre-clearing of lysate to minimize non-specific binding to beads

**Timing: 3 h**

The lysates obtained in the previous step are incubated with the streptavidin-coated magnetic beads to remove non-specific binding of proteins and RNA to the beads.

20. Thaw the frozen lysates from the step “cell lysis” at 22 °C.
21. Resuspend the beads from the “preparation of beads” step and aliquot 100 μl of beads suspension per pulldown for each condition into a new DNA LoBind 15 ml tube.

***Note:*** Create two tubes, one for each condition (mock versus infected), each with 600 μl of beads.

22. Separate the beads on the DynaMag™-15 Magnet and remove the supernatant.

***Note:*** Within the next step, the pre-clear is performed in a pooled manner for each condition (mock or infected). In the subsequent steps, the pre-cleared lysate of each condition is separated into six equal volumes to set-up six individual pulldowns (corresponding to vtRNA1-1, vtRNA1-2, vtRNA1-3, and for each a sense and antisense pulldown).

23. Add the thawed lysates from the two conditions (mock or infected) to the beads and resuspend the beads through gentle pipetting.
24. Incubate the lysate in a thermomixer with intermittent mixing at 1100 rpm (30 sec on, 30 sec off) at 22 °C.
25. Separate the supernatant from the beads (which is the pre-cleared lysate) through the DynaMag™-15 Magnet.
26. Remove an aliquot from each condition (mock and infected) to serve as “input” control, take 45 μl for Western blot and 20 μl for RNA isolation.

***Note:*** Collect the samples for RNA isolation in a PCR strip for the proteinase K treatment during RNA elution and store at −80°C. Dilute the samples for western blot analysis with 4x protein sample buffer and store at −20°C.

27. Divide the supernatant over the appropriate number of tubes, i.e., the number of pulldowns per condition.

***Note:*** For this experimental set-up, each condition is subdivided in 6 pulldowns, i.e. a sense and antisense pulldown for each vtRNA.

### Hybridization, capture of the hybridized RNA, and protein elution

**Timing: 8 h**

The pre-cleared lysates are incubated with the hybridization probe that binds the vtRNA. The hybridized vtRNA-protein complexes are retrieved using streptavidin-coated magnetic beads and subsequently eluted from the beads.

28. Denature the appropriate amount of probe (300 pmol of probe per 3 mg of protein per pulldown) by heating at 85 °C for 3 min in a thermocycler. Afterwards, immediately move the probes onto ice.

**CRITICAL:** The amount of probe used for the pulldown should be titrated to achieve the optimal probe concentration to retrieve the target of interest, as discussed in “Problem 2”.

29. Add the probe to the lysate and incubate for 2 hours at 67 °C with intermittent mixing at 1100 rpm (30s on, 30s off).

***Note:*** After incubation, an RNA sample can be taken to validate the integrity of the RNA through Bioanalyzer analysis.

30. Remove the heat block from the thermocycler to allow the samples to slowly cool down to RT (approximately 1 hour).
  a. In the meantime, take the streptavidin-coated beads prepared during the “Preparation of beads to capture the probe” step and divide 200 μl of beads into a new 15ml DNA LoBind tube.
    ***Note:*** Prepare one tube for each pulldown.
  b. Remove the supernatant from the beads through separation with the DynaMag™-15 prior to the addition of the lysate to the beads.
31. After the 1 hour cooldown period (and prior to addition to the beads), remove a 20 μl aliquot from each individual sample (resulting in 12 samples in total), serving as ‘input with probe sample’ for downstream qPCR analysis.

***Note:*** Collect the samples for RNA isolation in a PCR strip for the proteinase K treatment during RNA elution and store at −80°C.

32. Subsequently, add the lysates to the beads and mix through gentle pipetting.
33. Incubate for 1 hour at RT with intermittent mixing at 1100 rpm in a thermomixer (30s on, 30s off).
34. Separate the beads with the DynaMag™-15 and take an aliquot of “unbound sample” from the supernatant for each lysate, as described in step 4 (45 μl for Western blot and 20 μl for RNA isolation). Discard the leftover supernatant.

***Note:*** Collect the samples for RNA isolation in a PCR strip for the proteinase K treatment during RNA elution and store at −80°C. Dilute the samples for western blot analysis with 4x protein sample buffer and store at −20°C.

35. Wash the beads 6 times with 0.5 mL 1x Hybridization buffer using the DynaMag™-15 magnet.
36. During the last wash step, take an aliquot serving as “pulldown sample” from the bead suspension (30 μl for Western blot, 5 μl for RNA isolation), as described in step 4.

**CRITICAL:** These samples should be taken from the lysates directly after thorough vortexing, to ensure equal distribution of the beads in the lysate.

***Note:*** Collect the samples for RNA isolation in a PCR strip for the proteinase K treatment during RNA elution and store at −80°C. Dilute the samples for western blot analysis with 4x protein sample buffer and store at −20°C.

37. Next, remove the supernatant from the remaining beads using the magnet and discard the supernatant.
38. Resuspend the beads in 100 μl Benzonase Elution buffer and add 0.5 μl of Benzonase (125 U) to each tube.

***Note:*** This step ensures the elution of the vtRNA-protein complexes from the beads by degrading the hybridization probe.

39. Incubate for 2 hours at 37 °C with intermittent mixing at 1100 rpm on a thermomixer (30s on, 30s off).
40. Separate the beads on the DynaMag™-15 magnet and transfer the supernatant to a fresh DNA LoBind tube. Freeze samples at −80 °C.

***Note:*** Eluted proteins can be stored at −80°C for a maximum of 1 month.

### Preparation of the protein samples for analysis by mass spectrometry

**Timing: 4 h**

In this step, the samples are further processed through freeze-drying to remove the elution buffer and to concentrate the samples. Subsequently, the samples are resolved on an SDS-PAGE gel, extracted, and digested with trypsin to prepare for mass spectrometry.

41. Freeze-dry the samples for 1-2 hours in a freeze-dryer.

***Note:*** Drying can be accelerated by freezing the samples in liquid nitrogen prior to freeze-drying.

42. Resuspend the dried pellet in 16 μl UltraPure water (the volume of the leftover pellet is estimated to be approximately 2 μl).
43. Add 6 μl of 4X protein sample buffer and boil at 95 °C for 5 minutes.
44. Resolve the proteins on a 4–15% Mini-PROTEAN® TGX™ Precast Protein Gels gel at 80 V until the samples have run approximately 1 cm into the gel.

***Note:*** Use pre-cast gels to minimize contamination of the samples.

***Note:*** Leave an empty lane between each sample to avoid cross-contamination.

***Note:*** Load only protein samples corresponding to the sense and antisense pulldown on the Tris-Glycine gel. The protein samples taken as “input”, “unbound” and “pulldown” are stored and may be analyzed at a later stage for quality control purposes (e.g. to verify equal protein concentration/protein expression across conditions).

45. Fix the gel using the SilverQuest silver staining kit according to manufacturer’s protocol.

***Note:*** It is possible that the SilverQuest silver stain does not reveal an enrichment of any proteins when comparing the sense (negative control) pulldown and the antisense pulldown, as discussed in “Problem 3”.

46. Divide each sample lane in 5 slices, cut each slice further into small blocks.
47. Next, wash with PBS and reduce the samples with 10 mM dithiothreitol and subsequently alkylate them with 50 mM of iodoacetamide.
48. Perform in-gel trypsin digestion (12.5 ng/μl of trypsin) for 16 hours at 37 °C with the Proteineer DP digestion robot.
49. Extract tryptic peptides from the individual slices and subsequently lyophilize them with a freeze-dryer.

***Note:*** Lyophilized peptides can be stored at −80°C for a maximum of 1 month prior to mass spectrometry.

50. Resuspend the samples in water/formic acid (100/0.1% (v/v)) and subject the samples to mass spectrometry analysis.
51. Load the samples on a HPLC MS/MS system with a Ultimate3000 nano gradient combined with the Exploris480 mass spectrometer.

***Note:*** A cartridge precolumn (300 μm × 5 mm, C18 PepMap, 5 μm, 100 A) is used within the mass spectrometer.

***Note:*** Elution is performed with a homemade analytical nano-HPLC column (50 cm × 75 μm; Reprosil-Pur C18-AQ 1.9 μm, 120 A).

***Note:*** A more detailed description of the procedure can be found in Stok et al., 2025^1^ and Guttman and McHugh., 2018.^9^

### Preparation of the RNA samples to measure pull-down efficiency

**Timing: 2 h**

In this step, the eluted RNA obtained during the last step of the pulldown is extracted from the beads by performing SILANE bead-based RNA extraction, alongside the “*input*” and *“unbound”* samples. These samples are then analyzed by qPCR as described in the step “RNA extraction, cDNA synthesis and qPCR”. Alternatively, the RNA samples obtained in this step can be directly used in Northern blot analysis tailored to small RNAs.^13^

52. Separate the supernatant of the RNA elution sample through the DynaMag™-2 Magnet and resuspend the beads in 20 μl NLS Elution buffer.
53. Heat the samples for 2 minutes at 95 °C.
54. Separate the beads through the DynaMag™-2 Magnet and transfer the supernatant (containing the RNA) to a new PCR tube.
55. Equalize the volumes of all RNA samples with NLS Elution buffer.

***Note:*** Also thaw the input and unbound samples that were obtained earlier to perform Proteinase K digestion on these samples.

56. Incubate the RNA samples with 1 mg/ml of proteinase K and incubate at 55 °C for 1 hour.

***Note:*** Samples can be frozen at −80°C for 1 month until SILANE clean-up.

***Note:*** Based on the selected pulldown validation method, the RNA samples can be further processed in the step “RNA extraction, cDNA synthesis and qPCR” or be analyzed by Northern blot.

### RNA extraction, cDNA synthesis, and qPCR

**Timing: 3 h**

In this step, the RNA aliquots taken at different steps of the protocol are isolated through SILANE bead-based RNA extraction and analysed through qPCR.

57. Thaw the proteinase K-treated RNA samples on ice.
  a. In the meantime, aliquot 20 μl of Dynabeads™ MyOne™ Silane beads for each RNA isolation.
  b. Separate the storage buffer from the beads through the DynaMag™-PCR Magnet and resuspend the beads in 60 μl RLT buffer.
58. Transfer the resuspended beads (60 μl) to the 20 μl RNA samples and mix well.
59. Add 120 μl of 100% EtOH and incubate for two minutes to 22 °C to allow the RNA to bind to the beads.
60. Remove the supernatant through magnetic separation with the DynaMag™-PCR Magnet and wash the beads twice with 200 μl 100% EtOH.
61. After removal of the second wash step, airdry the samples for approximately 5 minutes.
62. Elute the RNA in 20 μl TURBO DNase elution buffer by incubation for 20 minutes at 37 °C.
63. Perform a secondary SILANE bead-based RNA extraction, exactly as described above but elute the RNA in 10 μl UltraPure water.

***Note:*** RNA can be stored at −80°C to continue with cDNA synthesis when desired.

64. Continue by synthesizing cDNA from the isolated RNA (using 5 μl of RNA as input) using the SuperScript IV VILO Master Mix.

***Note:*** For this step, any commercially available cDNA synthesis kit can be used.

***Note:*** No DNase step is necessary, as the RNA has already been treated with TURBO DNase.

65. Perform qPCR on the cDNA samples to validate productive viral infection, vtRNA upregulation, and pulldown efficiency (if applicable) as described in “Quality control considerations” using vtRNA-specific primers, PowerUp™ SYBR™ Green Master Mix and a QuantStudio 3 qPCR machine (or equivalent), according to manufacturer’s instructions.

***Note:*** Any commercially available qPCR mastermix and qPCR machine can be used in this step.

## Expected outcomes

The first dataset generated within this protocol is obtained in the step: “Preparation of the RNA samples to measure pulldown efficiency”, which results in data validating the vtRNA pulldown, either through qPCR or Northern blot, as described earlier within the protocol.

The second dataset is the raw mass spectrometry data, which yields a list of peptide counts and coverage, from which the protein identity can determined, as described in the section “quantification and statistical analysis” underneath.

## Quantification and statistical analysis

The raw dataset obtained through mass spectrometry analysis is first converted from peptide hits to a peak list (containing the mass, charge and intensity of each peptide fragment) using Proteome Discoverer software. This list is subsequently submitted to the minimal UniProt database, supplemented with the protein sequences from the EMCV polyprotein or other relevant viral protein sequences to allow for reference to the viral genome. Protein identification is carried out using Mascot, allowing a mass tolerance of 10 ppm for precursor ions (maximum deviation of theoretical mass of intact peptides measured before fragmentation) and 0.02 Da for fragment ions (maximum deviation of the theoretical fragmented ions generated through digestion). These parameters are applied assuming trypsin as the digestion enzyme. Further sub-settings include methionine oxidation and acetylation as variable modifications, while carbamidomethyl is set as fixed modification of cysteines. The false-discovery rate is set at < 1%.

Subsequent filtering has to be performed to remove common contaminants such as benzonase, BSA and common skin proteins (for example keratin). In addition, we recommend to exclude proteins with less than 2 unique peptides from the dataset to decrease the number of false positives.

**Figure 1:**
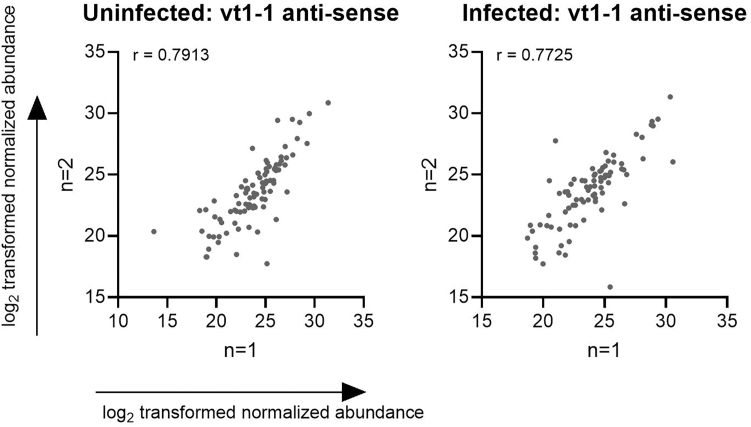
Reproducibility between RAP-MS replicates Proteins identified in the antisense pulldown of vtRNA1-1 are plotted for each replicate experiment on different axes with a log-transformed relative abundance. The Pearson correlation coefficient between the replicate experiments was calculated and displayed as r value.

Follow-up analysis regarding the quantification and visualization of the individual protein hits is performed with Perseus software. Stratify the data per individual vtRNA and based on experimental conditions (i.e., mock *versus* infected cells). We recommend to perform RAP-MS in duplicate, and preferably triplicate, experiments. Only proteins that are identified in all replicate experiments should be included in downstream analysis. As a quality control of the reproducibility of the data a Pearson correlation coefficient can be calculated (Figure 1). Ideally, a strong correlation for the “hits” should be observed between two replicate experiments. The approximate enrichment of vtRNA-specific interactors is measured by calculating the fold change between the antisense pulldown (vtRNA1-1, vtRNA1-2 or vtRNA1-3) and the respective sense pulldown (negative control), by comparing the log-corrected and normalized intensities from the antisense pulldown to the log-corrected normalized proteins in the sense pulldown. It is not uncommon that the obtained vtRNA interactomes share a high degree of similarity across the different vtRNAs. Furthermore, the proteins retrieved with the sense probe may have a certain degree of overlap with the proteins isolated with the anti-sense probe. Solutions to these two issues are discussed in “Problem 4” and “Problem 5”, respectively. The analyzed dataset is attached in the supplementary data, while the raw dataset containing the peptide counts and coverage can be found in Stok et al., 2025.^1^

## Limitations

A limitation of the provided protocol is the fact that the designed probes are not entirely unique for its target vtRNA. This is attributable to the high degree of homology between vtRNA1-1, vtRNA1-2 and vtRNA1-3. While this can be circumvented in the case of long ncRNAs by using a tiled probe approach, in which multiple probes of 90 nt long are employed, this is not feasible for vtRNAs due to their limited length. The individual probes may therefore also interact to some degree with other vtRNA family members. In practice, this means that some of the identified interactors may be a shared interactor of the vtRNA *family*. In addition, as discussed under ‘Probe design’, the use of *control* probes that are the reverse complement to the *target* probe bear the risk to unintentionally retrieve proteins that interact with intramolecular base-paired regions within vtRNAs, leading to potential exclusion of false negatives. To circumvent this, we recommend the use of an additional, non-related control probe. In addition, RAP-MS should be coupled to a protein-centric method to validate the identified RNA-protein interactions, such as RIP-qPCR, which involves the immunoprecipitation of a protein of interest through specific antibodies, followed by RNA extraction and qPCR analysis for the transcripts of interest.

## Troubleshooting

### Problem 1

Low amount of protein yield from the EMCV-infected dishes, related to the step “cell lysis”. EMCV is a strongly lytic virus, which can impact on the protein yield from the infected conditions. The input lysate should contain at least 3 mg of protein for each pulldown to ensure appropriate coverage in downstream mass spectrometry analysis.

### Potential solution

This problem can be solved by careful optimization of the MOI and infection time to find the correct balance between the number of infected cells and the severity of infection, i.e., the cytopathic effect. In addition, a greater number of dishes may be required for this condition to eventually yield > 3 mg of protein lysate.

### Problem 2

The pulldown of the target of interest (i.e., vtRNA) is inefficient, related to the step “Hybridization, capture of the hybridized RNA, and protein elution”. This can be caused by a suboptimal probe concentration.

### Potential solution

We recommend to perform a titration experiment to compare different amounts of probes. The amount of required probe is highly dependent on the expression of the target of interest. Therefore, to determine the optimal probe concentration, a small-scale titration experiment should be performed to determine the optimal probe concentration. Note that the amount of probe should never exceed the capacity of the beads. To determine the pulldown efficiency in such a titration experiment, we recommend to analyze the retrieval of a known protein interactor of the transcript of interest (e.g., TEP1 in the case of vtRNAs). Alternatively, if there are no known interactors, qPCR or Northern blot analysis can be performed on the pulldown to determine the optimal probe concentration that yields the most efficient retrieval of the target of interest.

### Problem 3

No differences are observed in the banding pattern when comparing the antisense pulldown and the sense pulldown by silver staining, related to the step “Preparation of the protein samples for analysis by mass spectrometry”.

### Potential solution

The SilverQuest Silver Staining kit provides a highly sensitive protein stain. However, common (background) proteins such as BSA, keratin and benzonase are always found as contaminants in mass spectrometry samples. These proteins may overshadow the small fraction of proteins that are UV-crosslinked and specifically pulled down, rendering it difficult to visualize specific vtRNA-interacting proteins through SDS-PAGE and silver stain analysis when comparing the antisense and the sense pulldown. As this necessarily an unexpected result but rather a limitation of the technique, no readjustment of the protocol is necessary.

### Problem 4

The protein interactomes of the different vtRNAs show a high degree of similarity amongst the different vtRNAs, related to the step “quantification and statistical analysis”.

As indicated, vtRNAs have a highly homologous structure, resulting in complementarity of the antisense vtRNA probes to different vtRNAs.

### Potential solution

To validate the interaction of a specific vtRNA with a protein identified through RAP-MS, a protein-centric method can be employed, such as RIP-qPCR. Following immunoprecipitation of candidate interactors, co-precipitating RNAs can be analyzed by qPCR for enrichment of vtRNAs compared to a control IP. This is an complementary method to verify the findings in the RAP-MS.

### Problem 5

The sense (control) probe retrieves of lot of proteins that are also bound by the antisense probe, related to the step “quantification and statistical analysis”.

As a control, the complementary sequence of the antisense probes is used (i.e., the sense probe). However, due to the secondary structure and intramolecular complementarity of the vtRNAs these sense probes may to some extent bind to (part of) the vtRNA for which they are meant to serve as a negative control. This results in the potential retrieval of bona fide vtRNA interactors via the negative control and the exclusion of such ‘false negatives’ from downstream analysis.

### Potential solution

An alternative control probe could be used, for example a scrambled version of the antisense probe.

## Resource availability

### Lead contact

Questions and requests regarding resources and reagents should be directed to and will be fulfilled by the lead contact, Annemarthe G. van der Veen (a.g.van_der_veen@lumc.nl).

### Technical contact

Technical questions on executing this protocol should be directed to and will be answered by the technical contact, Annemarthe G. van der Veen (a.g.van_der_veen@lumc.nl).

### Materials availability

This study did not generate new, unique reagents.

### Data and code availability

The analyzed RAP-MS dataset is included as supplemental data with this manuscript. The raw mass spectrometry proteomics data have been deposited to the ProteomeXchange Consortium via the PRIDE partner repository with the dataset identifier PXD055065.

## Acknowledgments

We are grateful to Peter A. van Veelen, Rayman T.N. Tjokrodirijo, and Arnoud H. de Ru for help with mass spectrometry analysis. We thank all members of the van der Veen group for critical discussions. This work was supported by a research grant from the Institute for Chemical Immunology (ICI-00203), which is funded by a Gravitation project (024.002.009) from the Netherlands Organization for Scientific Research (NWO), a VIDI research grant (09150171910070), an Aspasia award (015.015.046), and an ENW-M1 grant (OCENW.M.22.467) from the NWO, and the LUMC.

## Author contributions

Methodology, S.B.v.d.K., J.E.S., and A.G.v.d.V.; investigation, S.B.v.d.K. and J.E.S.; writing, S.B.v.d.K.; review and editing, J.E.S. and A.G.v.d.V.; funding acquisition, A.G.v.d.V.; supervision, A.G.v.d.V.

## Declaration of interests

The authors declare no competing interests.

## Supplemental information

Data S1. Example analyzed RAP-MS dataset, related to Data and Code Availability

## References

[1] Stok J.E., van der Kooij S.B., Gravekamp D., ter Haar L.R., de Wolf J.W., Salgado-Benvindo C., Nelemans T., Grijmans B.J.M., Tjokrodirijo R.T.N., de Ru A.H. (2025). Vault RNAs aid RNA virus infection by facilitating cytoplasmic localization of hnRNP C and ELAVL1. Cell Rep..

[2] Stadler P.F., Chen J.J.L., Hackermüller J., Hoffmann S., Horn F., Khaitovich P., Kretzschmar A.K., Mosig A., Prohaska S.J., Qi X. (2009). Evolution of Vault RNAs. Mol. Biol. Evol..

[3] Büscher M., Horos R., Huppertz I., Haubrich K., Dobrev N., Baudin F., Hennig J., Hentze M.W. (2022). Vault RNA1–1 riboregulates the autophagic function of p62 by binding to lysine 7 and arginine 21, both of which are critical for p62 oligomerization. RNA.

[4] Horos R., Büscher M., Kleinendorst R., Alleaume A.-M., Tarafder A.K., Schwarzl T., Dziuba D., Tischer C., Zielonka E.M., Adak A. (2019). The Small Non-coding Vault RNA1-1 Acts as a Riboregulator of Autophagy. Cell.

[5] Bracher L., Ferro I., Pulido-Quetglas C., Ruepp M.-D., Johnson R., Polacek N. (2020). Human vtRNA1-1 Levels Modulate Signaling Pathways and Regulate Apoptosis in Human Cancer Cells. Biomolecules.

[6] Li F., Chen Y., Zhang Z., Ouyang J., Wang Y., Yan R., Huang S., Gao G.F., Guo G., Chen J.-L. (2015). Robust expression of vault RNAs induced by influenza A virus plays a critical role in suppression of PKR-mediated innate immunity. Acids Res.

[7] Hafner M., Katsantoni M., Köster T., Marks J., Mukherjee J., Staiger D., Ule J., Zavolan M. (2021). CLIP and complementary methods. Nat. Rev. Methods Primers.

[8] McHugh C.A., Chen C.-K., Chow A., Surka C.F., Tran C., McDonel P., Pandya-Jones A., Blanco M., Burghard C., Moradian A. (2015). The Xist lncRNA interacts directly with sharp to silence transcription through HDAC3. Nature.

[9] Ramanathan M., Porter D.F., Khavari P.A. (2019). Methods to study RNA–protein interactions. Nat. Methods.

[10] McHugh C.A., Guttman M. (2018). Rap-MS: A method to identify proteins that interact directly with a specific RNA molecule in cells. Methods Mol. Biol..

[11] Lee S., Lee Y.-s., Choi Y., Son A., Park Y., Lee K.-M., Kim J., Kim J.-S., Kim V.N. (2021). The SARS-CoV-2 RNA interactome. Mol. Cell.

[12] Gagliardi M., Matarazzo M.R., Lanzuolo C., Bodega B. (2016).

[13] Rio D.C. (2014). Northern Blots for Small RNAs and MicroRNAs. Cold Spring Harb. Protoc..

